# Supplementary material for: Exploring the Molecular Mechanisms of Macrolide Resistance in Laboratory Mutant Helicobacter pylori
Source: Antibiotics (Basel). 2024 Apr 26;13(5):396. doi: 10.3390/antibiotics13050396 (PMC11117244; doi:10.3390/antibiotics13050396)
Supplement: Supplementary file 1 [file antibiotics-13-00396-s001.zip › antibiotics-2918124-supplementary.pdf]

Supplementary Materials

Exploring the Molecular Mechanisms of Macrolide Resistance in Laboratory Mutant *Helicobacter pylori*

Meltem AYAŞ[1,2]*, Sinem ÖKTEM OKULLU[3], Orhan ÖZCAN[4], Tanıl KOCAGÖZ[2,3], Yeşim GÜROL[3]

[1] Department of Medical Laboratory Techniques, Vocational School of Health Services, Acibadem Mehmet Ali Aydinlar University, Istanbul, Turkey; meltem.ayas@acibadem.edu.tr.

[2] Department of Medical Biotechnology, Institute of Health Sciences, Acibadem Mehmet Ali Aydinlar University, Istanbul, Turkey; tanilkocagoz@gmail.com.

[3] Department of Medical Microbiology, Faculty of Medicine, Acıbadem Mehmet Ali Aydınlar University, Istanbul, Turkey; sinem.oktem@acibadem.edu.tr , yesim.gurol@acibadem.edu.tr.

[4] TrioScience Biotechnology, Istanbul, Turkey; orhn.ozcn@hotmail.com.

* Correspondence: meltem.ayas@acibadem.edu.tr

Table S1. The combined bioinformatics analysis results of *H. pylori* G27 and Mutant-1 whole genome data.

CHROM	POS	REF	ALT	Codes For	Gene	aa change
NC_011333_G27	7125	GT	G	NA(non-coding region)	NA	NA
NC_011333_Mutant1	7125	GT	G	NA	NA	NA
NC_011333_Mutant1	20317	C	CTTTAATTAAG	NA	NA	NA
NC_011333_G27	74846	T	TA	urease accessory protein UreF CDS	Pseudogene	Not a pseudogene
NC_011333_Mutant1	74846	T	TA	urease accessory protein UreF CDS	Pseudogene	Not a pseudogene
NC_011333_G27	156870	A	AT	hypothetical protein CDS	Pseudogene	Not a pseudogene
NC_011333_Mutant1	156870	A	AT	hypothetical protein CDS	Pseudogene	Not a pseudogene
NC_011333_G27	204054	T	TG	hypothetical protein CDS	Pseudogene	Not a pseudogene
NC_011333_Mutant1	204054	T	TG	hypothetical protein CDS	Pseudogene	Not a pseudogene
NC_011333_Mutant1	210816	G	GT	NA(non-coding region)	NA	NA
NC_011333_G27	232310	G	GA	NA(non-coding region)	NA	NA
NC_011333_Mutant1	232310	G	GA	NA(non-coding region)	NA	NA
NC_011333_G27	277049	A	AT	hypothetical protein CDS	Pseudogene	Not a pseudogene
NC_011333_Mutant1	277049	A	AT	hypothetical protein CDS	Pseudogene	Not a pseudogene
NC_011333_G27	277872	C	CA	hypothetical protein CDS	Pseudogene	Not a pseudogene
NC_011333_Mutant1	277872	C	CA	hypothetical protein CDS	Pseudogene	Not a pseudogene
NC_011333_G27	331419	G	GT	NA(non-coding region)	NA	NA
NC_011333_G27	334159	CT	C	tatA gene	Pseudogene	Not a pseudogene
NC_011333_Mutant1	334159	CT	C	tatA gene	Pseudogene	Not a pseudogene
NC_011333_Mutant1	355249	CAA	C	NA(non-coding region)	NA	NA
NC_011333_G27	401117	T	TG	hypothetical protein CDS	Pseudogene	Not a pseudogene
NC_011333_Mutant1	401117	T	TG	hypothetical protein CDS	Pseudogene	Not a pseudogene
NC_011333_G27	431781	A	AT	helix-turn-helix transcriptional regulator CDS	Pseudogene	Not a pseudogene
NC_011333_Mutant1	431781	A	AT	helix-turn-helix transcriptional regulator CDS	Pseudogene	Not a pseudogene
NC_011333_G27	433755	A	AG	outer membrane family protein CDS	Pseudogene	Not a pseudogene
NC_011333_Mutant1	433755	A	AG	outer membrane family protein CDS	Pseudogene	Not a pseudogene
NC_011333_G27	441037	C	CT	pgsA gene	Pseudogene	Not a pseudogene
NC_011333_Mutant1	441037	C	CT	pgsA gene	Pseudogene	Not a pseudogene
NC_011333_G27	507732	C	CT	NA(non-coding region)	NA	NA
NC_011333_Mutant1	507732	C	CT	NA(non-coding region)	NA	NA
NC_011333_G27	508421	A	AC	NA(non-coding region)	NA	NA
NC_011333_Mutant1	508421	A	AC	NA(non-coding region)	NA	NA
NC_011333_G27	523568	G	GT	cag pathogenicity island protein CDS	Pseudogene	Not a pseudogene
NC_011333_Mutant1	523568	G	GT	cag pathogenicity island protein CDS	Pseudogene	Not a pseudogene
NC_011333_G27	551897	G	GT	CagA		
NC_011333_Mutant1	551897	G	GT	CagA		
NC_011333_G27	555760	G	GA	rsmI gene	Pseudogene	Not a pseudogene
NC_011333_Mutant1	555760	G	GA	rsmI gene	Pseudogene	Not a pseudogene
NC_011333_G27	566958	A	AT	NA(non-coding region)	NA	NA
NC_011333_Mutant1	566958	A	AT	NA(non-coding region)	NA	NA
NC_011333_G27	581541	CT	C	energy transducer TonB CDS	Pseudogene	Not a pseudogene
NC_011333_Mutant1	581541	CT	C	energy transducer TonB CDS	Pseudogene	Not a pseudogene
NC_011333_G27	581557	CT	C	energy transducer TonB CDS	Pseudogene	Not a pseudogene
NC_011333_Mutant1	581557	CT	C	energy transducer TonB CDS	Pseudogene	Not a pseudogene
NC_011333_G27	581570	CT	C	energy transducer TonB CDS	Pseudogene	Not a pseudogene
NC_011333_Mutant1	581570	CT	C	energy transducer TonB CDS	Pseudogene	Not a pseudogene
NC_011333_Mutant1	657913	G	GC	NA(non-coding region)	NA	NA
NC_011333_Mutant1	694384	A	G	sulfite exporter TauE/SafE family protein CDS	sulfite exporter	L>P

Table S1. The combined bioinformatics analysis results of *H. pylori* G27 and Mutant-1 whole genome data (continued)

CHROM	POS	REF	ALT	Codes For	Gene	aa change
NC_011333_G27	725944	T	TA	uvrA	Pseudogene	Not a pseudogene
NC_011333_Mutant1	725944	T	TA	uvrA	Pseudogene	Not a pseudogene
NC_011333_G27	739196	CG	C	DNA polymeras	Pseudogene	Not a pseudogene
NC_011333_Mutant1	739196	CG	C	DNA polymeras	Pseudogene	Not a pseudogene
NC_011333_Mutant1	748242	CAA	C	NA(non-coding	NA	NA
NC_011333_Mutant1	758373	C	CA	NA(non-coding	NA	NA
NC_011333_Mutant1	834623	CTT	C	NA(non-coding	NA	NA
NC_011333_G27	936640	C	CA	NA(non-coding	NA	NA
NC_011333_G27	1028566	A	G	hypothetical protein CDS		huge frameshift
NC_011333_G27	1208733	CA	C	hopL CDS	Pseudogene	Not a pseudogene
NC_011333_Mutant1	1208733	CA	C	hopL CDS	Pseudogene	Not a pseudogene
NC_011333_G27	1228406	CA	C	NA(non-coding	NA	NA
NC_011333_Mutant1	1228406	CA	C	NA(non-coding	NA	NA
NC_011333_G27	1228626	A	AT	NA(non-coding	NA	NA
NC_011333_Mutant1	1228626	A	AT	NA(non-coding	NA	NA
NC_011333_Mutant1	1242247	T	C	DUF874 family	primary mutation distrupt gene than secondary mutations make gene not becoming psuedogene	TNQTPIQPKHLPNSKQPRSQR aa changes in final mutant
NC_011333_Mutant1	1242249	G	A			TNQTTTQPKHLPNSKQPHSQR aas in reference G27
NC_011333_Mutant1	1242264	G	T			
NC_011333_Mutant1	1242283	G	A			
NC_011333_Mutant1	1242287	T	G			
NC_011333_Mutant1	1251303	TAGGTTTTTTGGCGTCTTTTTTATCTTTGTGAGCCACTAATTGAG<br>AGCTTAACTCAGGTTTTTTGGCGTCTTTTTTATCTTTGTGAGCCA<br>CTAATTGAGAGCTTAACTCAGGTTTTTTGGCGTCTTTTTTATCTT<br>TGTGAGCCACTAATTGAGAGCTTAACTCAGGTTTTTTGGCGTCT<br>TTTTTATCTTTGTGAGCCACTAATTGAGAGCTTAACTCAGGTTT<br>TTTGGCGTCTTTTTTATCTTTGTGAGCCACTAATTGAGAGCTTA<br>ACTCAGGTTTTTTGGCGTCTTTTTTATCTTTGTGAGCCACTAAT<br>TGAGAGCTTAACTC	T	hypothetical pro	loss of a gene	
NC_011333_G27	1265037	C	CA	Name: DNA-dir	Pseudogene	Not a pseudogene
NC_011333_Mutant1	1265037	C	CA	Name: DNA-dir	Pseudogene	Not a pseudogene
NC_011333_G27	1304662	A	AT	alaS CDS	Pseudogene	Not a pseudogene
NC_011333_Mutant1	1304662	A	AT	alaS CDS	Pseudogene	Not a pseudogene
NC_011333_G27	1305908	CT	C	babB CDS	Pseudogene	Not a pseudogene
NC_011333_Mutant1	1305908	CT	C	babB CDS	Pseudogene	Not a pseudogene
NC_011333_G27	1333420	TA	T	NADH-quinone	Pseudogene	Not a pseudogene
NC_011333_Mutant1	1333420	TA	T	NADH-quinone	Pseudogene	Not a pseudogene
NC_011333_G27	1333463	GC	G	NADH-quinone	Pseudogene	Not a pseudogene
NC_011333_Mutant1	1333463	GC	G	NADH-quinone	Pseudogene	Not a pseudogene
NC_011333_G27	1333524	AT	A	NADH-quinone	Pseudogene	Not a pseudogene
NC_011333_Mutant1	1333524	AT	A	NADH-quinone	Pseudogene	Not a pseudogene
NC_011333_G27	1359849	A	AT	DNA-directed R	frameshifted at terminal ends	secondary mutation corrects error
NC_011333_Mutant1	1359849	A	AT	DNA-directed R	frameshifted at terminal ends	secondary mutation corrects error
NC_011333_G27	1367919	GT	G	NA(non-coding	NA	NA
NC_011333_Mutant1	1367919	GT	G	NA(non-coding	NA	NA
NC_011333_Mutant1	1392764	C	CA	NA(non-coding	NA	NA
NC_011333_G27	1410928	TA	T	4-hydroxybenzo	frameshifted at terminal ends	secondary mutation corrects error
NC_011333_Mutant1	1410928	TA	T	4-hydroxybenzo	frameshifted at terminal ends	secondary mutation corrects error
NC_011333_G27	1428286	G	GA	fliW CDS	frameshifted at terminal ends	secondary mutation corrects error
NC_011333_Mutant1	1428286	G	GA	fliW CDS	frameshifted at terminal ends	secondary mutation corrects error
NC_011333_G27	1480681	C	CT	rnpA CDS	frameshifted at 5' end	secondary mutation corrects error
NC_011333_Mutant1	1480681	C	CT	rnpA CDS	frameshifted at 5' end	secondary mutation corrects error
NC_011333_G27	1491244	T	TA	NA(non-coding	NA	NA
NC_011333_Mutant1	1491244	T	TA	NA(non-coding	NA	NA
NC_011333_G27	1491260	G	GA	NA(non-coding	NA	NA
NC_011333_Mutant1	1491260	G	GA	NA(non-coding	NA	NA
NC_011333_G27	1491283	GA	G	NA(non-coding	NA	NA
NC_011333_Mutant1	1491283	GA	G	NA(non-coding	NA	NA
NC_011333_G27	1541768	G	GA	heavy metal tra	Pseudogene	Not a pseudogene
NC_011333_Mutant1	1541768	G	GA	heavy metal tra	Pseudogene	Not a pseudogene
NC_011333_G27	1568738	T	TA	outer membran	Pseudogene	Not a pseudogene
NC_011333_Mutant1	1568738	T	TA	outer membran	Pseudogene	Not a pseudogene
NC_011333_Mutant1	1586573	CT	C	NA(non-coding	NA	NA
NC_011333_G27	1644852	AT	A	glycosyltransfer	in-silico SNP 6T repeat made Illumina sequencing error	
NC_011333_Mutant1	1644852	AT	A	glycosyltransfer	in-silico SNP 6T repeat made Illumina sequencing error	

Table S2. The combined bioinformatics analysis results of *H. pylori* G27 and Mutant-2 whole genome data

Position of Polymorphism	Details of Polymorphism
**1242247**	/Reference_Nucleotide(s)="T"<br>/Variant_Nucleotide(s)="C"<br>/Change="T -> C"<br>/Polymorphism_Type="Substitution"<br>/Qual="6569.77"<br>/Failed_Filters="PASS"<br>/Allele_count=2<br>/Allele_frequency=1<br>/Number_of_alleles=2<br>/Combined_Depth=150<br>/ExcessHet="3.0103"<br>/FS="0.000"<br>/MLEAC=2<br>/MLEAF=1<br>/Mapping_quality="42.39"<br>/QD="44.09"<br>/SOR="2.046"<br>/Genotype="1/1"<br>/AD="0,149"<br>/Sample_Depth=149<br>/Genotype_Quality=99<br>/PL="6598,448,0"<br>/label="C"
**1242249**	/Reference_Nucleotide(s)="G"<br>/Variant_Nucleotide(s)="A"<br>/Change="G -> A"<br>/Polymorphism_Type="Substitution"<br>/Qual="6569.77"<br>/Failed_Filters="PASS"<br>/Allele_count=2<br>/Allele_frequency=1<br>/Number_of_alleles=2<br>/Combined_Depth=139<br>/ExcessHet="3.0103"<br>/FS="0.000"<br>/MLEAC=2<br>/MLEAF=1<br>/Mapping_quality="40.84"<br>/QD="47.26"<br>/SOR="2.186"<br>/Genotype="1/1"<br>/AD="0,139"<br>/Sample_Depth=139<br>/Genotype_Quality=99<br>/PL="6598,448,0"<br>/label="A"
**1242264**	/Reference_Nucleotide(s)="G"<br>/Variant_Nucleotide(s)="T"<br>/Change="G -> T"<br>/Polymorphism_Type="Substitution"<br>/Qual="5731.77"<br>/Failed_Filters="MQFilter"<br>/Allele_count=2<br>/Allele_frequency=1<br>/Number_of_alleles=2<br>/Combined_Depth=116<br>/ExcessHet="3.0103"<br>/FS="0.000"<br>/MLEAC=2<br>/MLEAF=1<br>/Mapping_quality="38.00"<br>/QD="49.41"<br>/SOR="2.107"<br>/Genotype="1/1"<br>/AD="0,116"<br>/Sample_Depth=116<br>/Genotype_Quality=99<br>/PL="5760,385,0"<br>/label="T"

Table S2. The combined bioinformatics analysis results of *H. pylori* G27 and Mutant-2 whole genome data(continued)

**Position of Polymorphism**	**Details of Polymorphism**
**1242283**	/Reference_Nucleotide(s)="G"<br>/Variant_Nucleotide(s)="A"<br>/Change="G -> A"<br>/Polymorphism_Type="Substitution"<br>/Qual="6050.77"<br>/Failed_Filters="MQFilter"<br>/Allele_count=2<br>/Allele_frequency=1<br>/Number_of_alleles=2<br>/Combined_Depth=126<br>/ExcessHet="3.0103"<br>/FS="0.000"<br>/MLEAC=2<br>/MLEAF=1<br>/Mapping_quality="39.23"<br>/QD="48.02"<br>/SOR="1.953"<br>/Genotype="1/1"<br>/AD="0,126"<br>/Sample_Depth=126<br>/Genotype_Quality=99<br>/PL="6079,412,0"<br>/label="A"
**1242287**	/Reference_Nucleotide(s)="T"<br>/Variant_Nucleotide(s)="G"<br>/Change="T -> G"<br>/Polymorphism_Type="Substitution"<br>/Qual="5858.77"<br>/Failed_Filters="PASS"<br>/Allele_count=2<br>/Allele_frequency=1<br>/Number_of_alleles=2<br>/Combined_Depth=130<br>/ExcessHet="3.0103"<br>/FS="0.000"<br>/MLEAC=2<br>/MLEAF=1<br>/Mapping_quality="41.54"<br>/QD="45.07"<br>/SOR="2.452"<br>/Genotype="1/1"<br>/AD="0,130"<br>/Sample_Depth=130<br>/Genotype_Quality=99<br>/PL="5887,406,0"<br>/label="G"
**1243410**	/Reference_Nucleotide(s)="T"<br>/Variant_Nucleotide(s)="C"<br>/Change="T -> C"<br>/Polymorphism_Type="Substitution"<br>/Qual="2804.77"<br>/Failed_Filters="PASS"<br>/Allele_count=1<br>/Allele_frequency="0.5"<br>/Number_of_alleles=2<br>/BaseQRankSum="9.370"<br>/ClippingRankSum="0.000"<br>/Combined_Depth=429<br>/ExcessHet="3.0103"<br>/FS="2.013"<br>/MLEAC=1<br>/MLEAF="0.5"<br>/Mapping_quality="44.72"<br>/MQRankSum="-0.662"<br>/QD="6.54"<br>/ReadPosRankSum="-2.656"<br>/SOR="0.939"<br>/Genotype="0/1"<br>/AD="324,105"<br>/Sample_Depth=429<br>/Genotype_Quality=99<br>/PL="2833,0,10407"

Table S2. The combined bioinformatics analysis results of *H. pylori* G27 and Mutant-2 whole genome data(continued)

Position of Polymorphism	Details of Polymorphism
1243461	/Reference_Nucleotide(s)="C"<br>/Variant_Nucleotide(s)="T"<br>/Change="C -> T"<br>/Polymorphism_Type="Substitution"<br>/Qual="5134.77"<br>/Failed_Filters="PASS"<br>/Allele_count=1<br>/Allele_frequency="0.5"<br>/Number_of_alleles=2<br>/BaseQRankSum="-10.125"<br>/ClippingRankSum="0.000"<br>/Combined_Depth=693<br>/ExcessHet="3.0103"<br>/FS="4.063"<br>/MLEAC=1<br>/MLEAF="0.5"<br>/Mapping_quality="52.33"<br>/MQRankSum="-5.739"<br>/QD="7.46"<br>/ReadPosRankSum="2.685"<br>/SOR="1.079"<br>/Genotype="0/1"<br>/AD="488,200"<br>/Sample_Depth=688<br>/Genotype_Quality=99<br>/PL="5163,0,17376"
1243648	/Reference_Nucleotide(s)="G"<br>/Variant_Nucleotide(s)="A"<br>/Change="G -> A"<br>/Polymorphism_Type="Substitution"<br>/Qual="720.77"<br>/Failed_Filters="MQRankSumLow;QDFilter"<br>/Allele_count=1<br>/Allele_frequency="0.5"<br>/Number_of_alleles=2<br>/BaseQRankSum="-6.455"<br>/ClippingRankSum="0.000"<br>/Combined_Depth=971<br>/ExcessHet="3.0103"<br>/FS="47.951"<br>/MLEAC=1<br>/MLEAF="0.5"<br>/Mapping_quality="58.43"<br>/MQRankSum="-29.608"<br>/QD="0.74"<br>/ReadPosRankSum="-2.724"<br>/SOR="6.617"<br>/Genotype="0/1"<br>/AD="888,83"<br>/Sample_Depth=971<br>/Genotype_Quality=99<br>/PL="749,0,37701"
1243650	/Reference_Nucleotide(s)="G"<br>/Variant_Nucleotide(s)="A"<br>/Change="G -> A"<br>/Polymorphism_Type="Substitution"<br>/Qual="720.77"<br>/Failed_Filters="MQRankSumLow;QDFilter"<br>/Allele_count=1<br>/Allele_frequency="0.5"<br>/Number_of_alleles=2<br>/BaseQRankSum="3.239"<br>/ClippingRankSum="0.000"<br>/Combined_Depth=975<br>/ExcessHet="3.0103"<br>/FS="47.769"<br>/MLEAC=1<br>/MLEAF="0.5"<br>/Mapping_quality="58.46"<br>/MQRankSum="-29.838"<br>/QD="0.74"<br>/ReadPosRankSum="-3.160"<br>/SOR="7.403"<br>/Genotype="0/1"<br>/AD="893,81"<br>/Sample_Depth=974<br>/Genotype_Quality=99<br>/PL="749,0,37701"

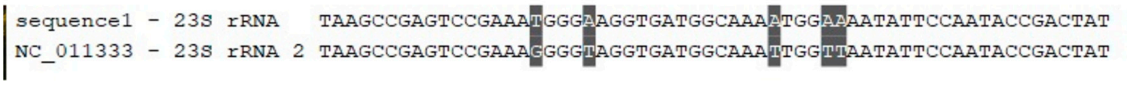

Figure S1. Position of SNPs detected (T1495A, T1494A, T1490A, T1476A, G1472T) via WGS in Mutant 1 strain.

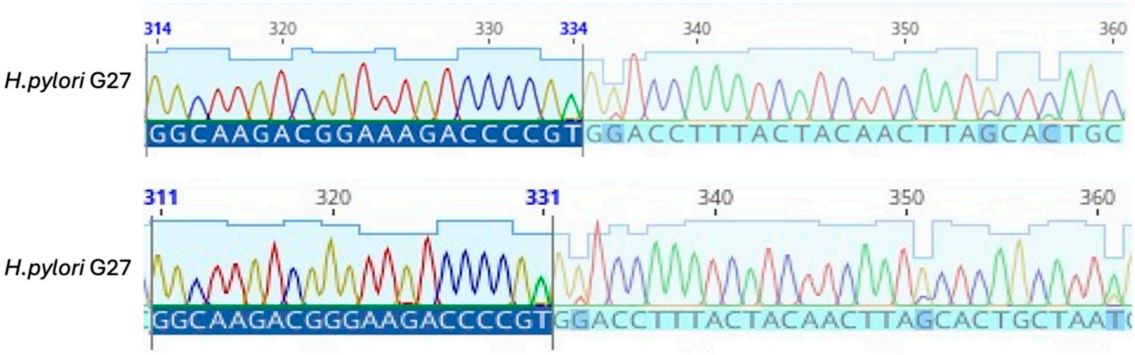

Figure S2: Signal decreases observed in Sanger sequence analysis are thought to be due to potential methylations occurring in the 23S rRNA region, leading to breaks.
